# Plasma glutathione peroxidase activity negatively correlates with atopic diseases in children but not adults: an exploratory study

**DOI:** 10.3389/falgy.2026.1771105

**Published:** 2026-03-12

**Authors:** Jennifer M. Pilat, Girish Hiremath, Joshua B. Wechsler, Evan S. Dellon, Yash A. Choksi, Matthew A. Buendia

**Affiliations:** 1 Division of Gastroenterology, Hepatology, and Nutrition, Department of Medicine, Vanderbilt University Medical Center, Nashville, TN, United States; 2 Division of Pediatric Gastroenterology, Hepatology, and Nutrition, Department of Pediatrics, Vanderbilt University Medical Center, Nashville, TN, United States; 3 Division of Gastroenterology, Hepatology, and Nutrition, Department of Pediatrics, Ann & Robert H. Lurie Children's Hospital of Chicago, Chicago, IL, United States; 4 Division of Gastroenterology and Hepatology, Department of Medicine, University of North Carolina at Chapel Hill School of Medicine, Chapel Hill, NC, United States; 5 Department of Research and Development, Veterans Affairs Tennessee Valley Health System, Nashville, TN, United States

**Keywords:** allergic rhinitis (AR), asthma, atopic dermatitis (AD), eosinophilic esophagitis (EoE), food allergy (FA), GPX: glutathione peroxidase

## Abstract

Atopic diseases, including atopic dermatitis, food allergy, allergic rhinitis, asthma, and eosinophilic esophagitis (EoE), are characterized by chronic inflammatory responses to aero- and/or food allergens. Oxidative stress has been increasingly implicated in the pathogenesis of these conditions. Antioxidant glutathione peroxidase (GPX) enzymes help mitigate oxidative stress by neutralizing hydrogen peroxide and lipid hydroperoxides. Since EoE requires invasive procedures for diagnosis and surveillance, our primary aim was to determine whether plasma GPX activity levels can be used as a non-invasive biomarker for disease activity, and our secondary aim was to determine whether plasma GPX activity levels correlate with other atopic diseases. While plasma GPX activity levels did not correlate with EoE, they did negatively correlate with non-EoE atopic disease in pediatric, but not adult subjects. These findings necessitate future studies to determine their clinical utility and underlying mechanisms.

## Introduction

Atopic diseases such as eczema, food allergy, allergic rhinitis, asthma, and eosinophilic esophagitis (EoE) share environmental and genomic risk factors, aero- and food allergen triggers, and immunological mediators and responses. While there are non-invasive approaches to identify other atopic diseases, diagnosis and monitoring of EoE requires repeated, invasive esophagogastroduodenoscopy (EGD) with biopsy, which is particularly burdensome for both children and their caregivers. Thus, there is an urgent unmet need for non-invasive EoE biomarkers to advance patient care.

Emerging evidence suggests that oxidative stress can enhance allergen penetration, promote epithelial damage, and perpetuate inflammatory cycles in EoE and other atopic diseases (1). Glutathione peroxidases (GPX1-8), a family of antioxidant enzymes that neutralize reactive oxygen species, have been shown to attenuate positive feedback loops between oxidative stress and inflammatory responses that characterize chronic immunoinflammatory diseases (2). Although GPX1, GPX2, GPX3, GPX4, GPX7, and GPX8 are all expressed in the esophagus, only *GPX3* was downregulated in esophageal biopsies from EoE patients (3). Importantly, GPX3 is the predominant GPX species in the plasma, and previous studies have shown that plasma GPX activity may serve as a non-invasive biomarker in patients with asthma and atopic dermatitis (4, 5). However, very little is known about plasma GPX activity in patients with EoE. To address this knowledge gap, we characterized plasma GPX activity primarily in EoE, and secondarily in other atopic diseases.

## Methods

Since EoE can affect all ages, we analyzed 36 pediatric (1−18 years) and 39 adult (≥18 years) samples collected at Lurie Children's Hospital (6) and the University of North Carolina (7), respectively. These samples were collected as part of ongoing Institutional Review Board-approved studies (#2011-14486, #22-385) registered on ClinicalTrials.gov (NCT05176249, NCT01988285). Informed consent (and assent, when applicable), including for use of stored samples in the future, was obtained. Race and ethnicity were self-reported. Upon collection, plasma samples were aliquoted, flash-frozen, and stored at −80 °C.

EoE was diagnosed according to 2011 consensus guidelines, which were standard of care at the time of sample collection (8). Specifically, those with active EoE had symptoms of esophageal dysfunction and a peak count of ≥15 eosinophils/high-power field (HPF) on esophageal biopsy after ≥8 weeks of proton pump inhibitor (PPI) therapy, with no other causes of esophageal eosinophilia. In the pediatric cohort, those with known EoE but <15 eosinophils/HPF on esophageal biopsy were classified as inactive EoE, and those without active or inactive EoE were classified as controls (7). In the adult cohort, controls had no upper endoscopic gastrointestinal inflammation, along with normal esophageal histology (6).

For the pediatric cohort, sample exclusion criteria included non-esophageal eosinophilic gastrointestinal disease (EGID), other inflammatory disease, infection, and chromosomal or anatomical abnormalities (6). For the adult cohort, sample exclusion criteria included EGIDs, known esophageal cancer, prior esophageal surgery, gastrointestinal bleeding, known esophageal varices, and medical instability or multiple comorbidities that precluded study enrollment, as judged clinically by the endoscopist (7). Additionally, no adult subjects used biologic drugs, systemic corticosteroids, other immunomodulators, or dietary (including antioxidant) supplements.

Endoscopic features of EoE were graded by the endoscopist per the Eosinophilic Esophagitis Endoscopic Reference Score (EREFS) system (9). Histological features of EoE were scored by experienced GI pathologists across ≥ 6 hematoxylin- and eosin-stained biopsies from both the distal and proximal esophagus. Both endoscopic and histological EoE severity were assessed using validated protocols to minimize interobserver variability (10, 11). Peak eosinophils were quantified per 0.23–0.24 mm^2^ HPF, and basal zone hyperplasia was classified as none (0), mild (1), mild-moderate (2), moderate (3), moderate-severe (4), or severe (5) in the pediatric cohort.

Plasma GPX activity was measured via an in-house enzyme-coupled assay as per (12). Briefly, 800 µL reaction cocktail [2.0 mM glutathione (Millipore Sigma, #G6529), 1 unit glutathione reductase (Millipore Sigma, #G3664), and 234 µM beta-nicotinamide dinucleotide phosphate (ThermoFisher Scientific, #J62089.MC)] and 100 µL single-use plasma sample aliquot (diluted 1:3 in water) were combined and incubated for 5 min at room temperature. Then, 100 µL of hydrogen peroxide (ThermoFisher Scientific, #18603250) was added for a final concentration of 250 µM, mixed again, and 340 nm absorbance was measured every 30 s over 5 min to quantify NADPH consumption as a readout of GPX activity. Water was used to calculate the lower limit of detection (1.4 nM NADPH • min^−1^) and background absorbance, which was subtracted from all sample measurements. All samples were assayed in technical duplicate.

Demographic and clinical features were characterized using descriptive statistics and compared among groups using one-way ANOVA (3 groups) or unpaired t-test (2 groups) for continuous variables, and Fisher's exact test for categorical variables in GraphPad Prism 10. Within each cohort, multivariable linear regression models were constructed in Stata 14.2, with plasma GPX activity as the dependent variable; EoE status (control/active/inactive), atopic status (EoE excluded, yes/no), and total atopic burden (EoE excluded) as independent variables; and age, sex, and PPI usage as control variables. The key assumptions of multivariable linear regression were assessed and satisfied as follows: independence (samples from unique individuals), linearity (between each independent and dependent variable on scatter plot), no multicollinearity (variance inflation factors of independent variables <5), homoscedasticity (constant variance across independent variables on residual plot), and normality without outliers (normally distributed data and residuals on histograms). Next, multivariable linear regression with Pearson's correlation was performed to assess relationships between GPX activity and atopic status. For all statistical analyses, two-tailed *p* < 0.05 was considered statistically significant.

## Results

**Figure 1:**
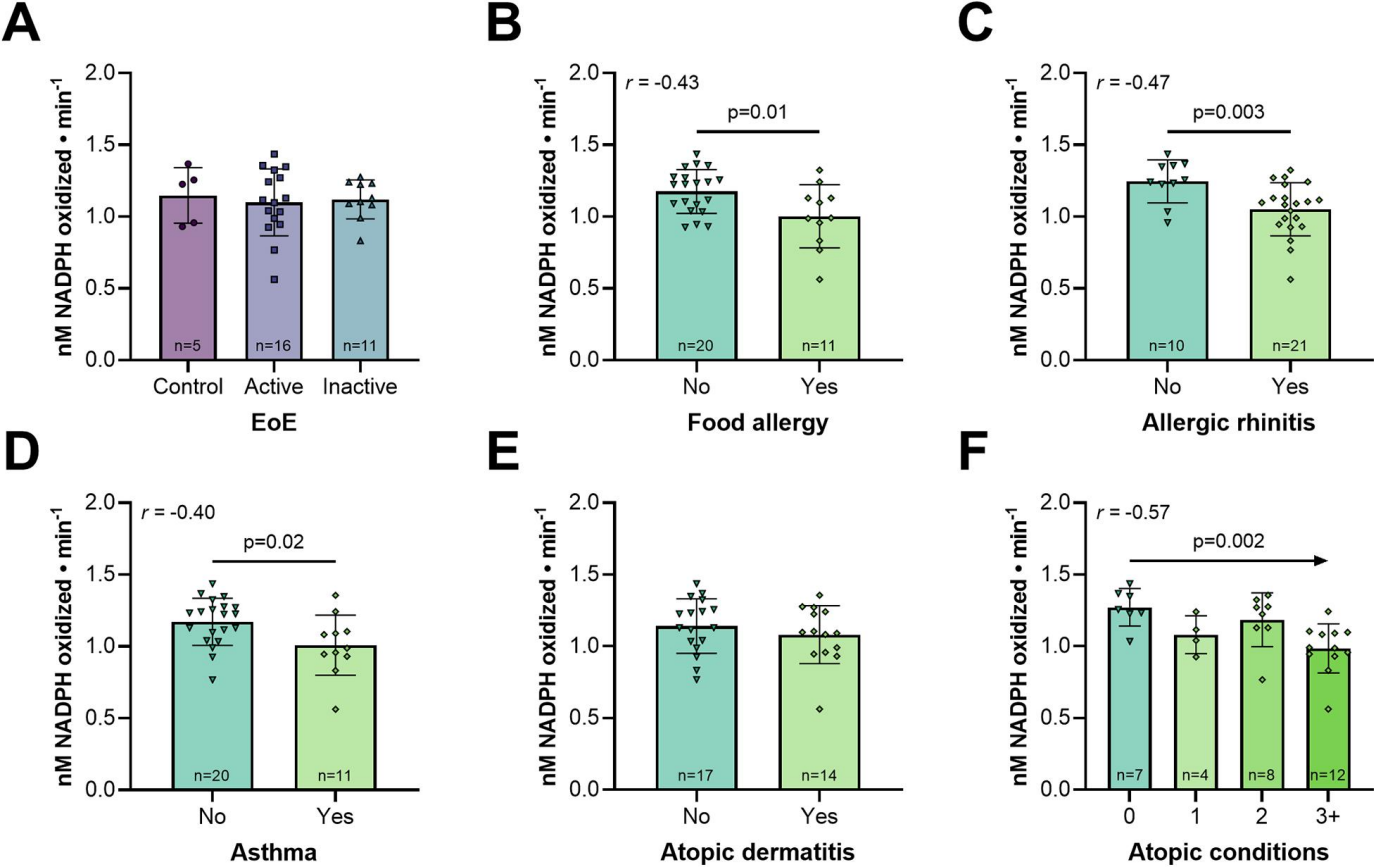
Lower plasma GPX activity correlates with non-EoE atopic diseases in pediatric subjects. Plasma GPX activity in patients **(A–E)** with **(A)** EoE, **(B)** food allergy, **(C)** allergic rhinitis, **(D)** asthma, or **(E)** atopic dermatitis; **(F)** stratified by number of atopic conditions (EoE excluded). Data are presented as mean ± SD. In **(F)**, each category only contains unique subjects. Multivariable linear regression with Pearson's correlation adjusted for age, sex, and proton pump inhibitor usage.

In the pediatric cohort, EoE was more prevalent in males, as well as more endoscopically and histologically severe in active EoE patients. Additionally, dietary elimination therapy was more common in inactive EoE patients. However, age, race, PPI therapy, atopic disease prevalence, and other gastrointestinal comorbidities were comparable among groups (Table 1). To robustly identify predictors of plasma GPX activity, we next performed multivariable linear regression analysis, with adjustments for age, sex, and PPI usage. Interestingly, plasma GPX activity did not correlate with EoE status (control: 1.15 ± 0.19, active: 1.10 ± 0.23, inactive: 1.12 ± 0.14; Figure 1A). We next interrogated relationships between plasma GPX activity and other atopic diseases across all subjects. Indeed, plasma GPX activity negatively correlated with food allergy (Figure 1B, Supplementary Figure S1A), allergic rhinitis (Figure 1C, Supplementary Figure S1B), and asthma (Figure 1D, Supplementary Figure S1C), but not atopic dermatitis (Figure 1E). Furthermore, plasma GPX activity negatively correlated with the cumulative burden of atopic conditions (Figure 1F, Supplementary Figure S1D).

**Table 1:** Demographic and clinical characteristics of pediatric subjects.

Characteristic	Control (*n* = 5)	Active EoE (*n* = 16)	Inactive EoE (*n* = 10)	*p* value
Age^a^	12.4 ± 5.3	11.3 ± 5.0	11.1 ± 5.1	0.890
Sex^b^				
Male	1 (20%)	15 (94%)	5 (50%)	0.002
Female	4 (80%)	1 (6%)	5 (50%)	
Race/Ethnicity^b^				
White, Non-Hispanic/Latino	2 (40%)	11 (68%)	8 (80%)	0.486
White, Hispanic or Latino	2 (40%)	2 (13%)	1 (10%)	
Other, Hispanic or Latino	0 (0%)	1 (6%)	1 (10%)	
Black	1 (20%)	2 (13%)	0 (0%)	
Endoscopic indices^a^ (EREFS score, 0–10)	0.6 ± 1.3	4.4 ± 2.6	0.8 ± 1.1	<0.001
Exudates (0–2)	0.2 ± 0.5	1.1 ± 0.8	0.2 ± 0.4	
Rings (0–3)	0.0 ± 0.0	0.7 ± 1.0	0.4 ± 0.7	
Edema (0–2)	0.2 ± 0.5	1.4 ± 0.6	0.1 ± 0.3	
Furrows (0–2)	0.2 ± 0.5	1.1 ± 0.7	0.1 ± 0.3	
Stricture (0–1)	0.0 ± 0.0	0.1 ± 0.3	0.0 ± 0.0	
Histological indices				
Peak eosinophils/HPF^a^	0.2 ± 0.5	58.4 ± 26.6	3.2 ± 4.3	<0.001
Basal zone hyperplasia (0–5)^a^	0.2 ± 0.5	3.1 ± 1.3	0.5 ± 0.5	<0.001
Therapeutic interventions				
Dietary elimination^b^	0 (0%)	0 (0%)	9 (90%)	<0.001
Proton pump inhibitors^b^	0 (0%)	8 (50%)	9 (90%)	0.087
Topical steroids	0 (0%)	0 (0%)	0 (0%)	N/A
Atopic disease^b^	2 (40%)	13 (81%)	9 (90%)	0.133
Atopic dermatitis	2 (40%)	5 (31%)	7 (70%)	
Food allergies	1 (20%)	7 (44%)	3 (30%)	
Allergic rhinitis	1 (20%)	12 (75%)	8 (80%)	
Asthma	2 (40%)	5 (31%)	4 (40%)	
Other gastrointestinal disease				
Gastroesophageal reflux disease^b^	0 (0%)	1 (6%)	2 (20%)	0.704
Inflammatory bowel disease^b^	0 (0%)	1 (6%)	0 (0%)	>0.999

HPF, high-power field (0.23 mm^2^). Continuous or categorical variables are reported as mean ± SD or frequency (percentage), respectively.

a One-way ANOVA.

b Fisher's exact test.

We next sought to determine whether our pediatric findings extended to adult subjects. As expected, adult EoE patients were younger and largely male, with more endoscopically and histologically severe disease compared to controls. Additionally, PPI therapy was more common in EoE than control subjects. However, we did not detect differences in race or atopic disease prevalence between controls and EoE patients (Table 2). As in the pediatric cohort, we next performed multivariable linear regression analysis, with adjustments for age, sex, and PPI usage. However, unlike in the pediatric cohort, plasma GPX activity did not correlate with EoE (control: 1.00 ± 0.10, EoE: 0.98 ± 0.20; Table 2) or other atopic diseases (without: 1.09 ± 0.20, with: 1.05 ± 0.20, *p* = 0.86).

**Table 2:** Demographic and clinical characteristics of adult subjects.

Characteristic	Control (*n* = 20)	EoE (*n* = 19)	*p* value
Age^a^	52.2 ± 13.1	38.2 ± 14.8	0.003
Sex^b^			
Male	6 (30%)	13 (68%)	0.026
Female	14 (70%)	6 (32%)	
Race^b^			
White	16 (80%)	18 (95%)	0.342
Black	4 (20%)	1 (5%)	
Endoscopic indices^a^ (EREFS score, 0–10)	0.8 ± 1.3	4.4 ± 1.8	<0.001
Exudates	0 (0%)	15 (79%)	
Rings	4 (20%)	16 (84%)	
Edema	2 (10%)	11 (58%)	
Furrows	2 (10%)	19 (100%)	
Stricture	6 (30%)	6 (32%)	
Histological indices			
Peak eosinophils/HPF^a^	0.3 ± 0.7	123.8 ± 49.7	<0.001
Therapeutic interventions			
Proton pump inhibitors^b^	7 (35%)	18 (95%)	<0.001
Atopic disease^b^	10 (50%)	9 (47%)	0.870
Atopic dermatitis	3 (15%)	2 (11%)	
Food allergies	5 (25%)	3 (16%)	
Allergic rhinitis	7 (35%)	8 (42%)	
Asthma	5 (25%)	5 (26%)	
Plasma GPX activity level^c^ (nM NADPH oxidized • min^−1^)	1.05 ± 0.22	0.99 ± 0.15	0.090

HPF, high-power field (0.24 mm^2^). Continuous or categorical variables are reported as mean ± SD or frequency (percentage), respectively.

a Unpaired t test.

b Fisher's exact test.

c Multivariable linear regression with Pearson's correlation adjusted for age, sex, and proton pump inhibitor usage.

## Discussion

Overall, the reductions in plasma GPX activity we observed in pediatric patients with atopic disease partially concur with previous, albeit very limited, studies. Like (4), we observed lower plasma GPX activity in pediatric asthma patients. However, unlike (5), we failed to observe lower plasma GPX activity in atopic dermatitis patients. Notably (5), enrolled subjects 10–65 years old and did not stratify results by age group, which complicates interpretation. Moreover, interassay variations in substrate choice and normalization methods confound direct comparisons of plasma GPX activity among studies.

To the best of our knowledge, we are the first to examine plasma GPX activity in EoE patients. As this study was retrospective and exploratory, we were unable to estimate effect size to perform formal power analysis. Thus, our inability to detect differences in plasma GPX activity between EoE and control subjects may represent type II error due to our small sample sizes, particularly in the pediatric control group, rather than true negative results. This constitutes a major limitation of this study. Future investigations warrant substantially larger sample sizes to increase power to detect potentially clinically relevant differences, especially in pediatric subjects.

When the clinical trials from which we obtained our samples began enrollment in 2011, then-current EoE diagnostic consensus criteria required failure of an 8-week PPI trial before diagnosis (8). This requirement was eliminated in 2018, when PPI-responsive EoE was formally recognized as a clinical EoE subphenotype (13). As such, all EoE patients in this study underwent PPI trial before sample collection; however, cumulative PPI exposure was unknown. Although PPIs largely function as acid suppressors, they can also function as antioxidants (14). Accordingly, PPIs may reduce systemic oxidative stress and thus affect plasma GPX activity. To address this potential confounder, we included ongoing PPI therapy as a control variable in all our regression models. However, prior PPI exposure duration may have affected plasma GPX activity.

Additionally, we did not collect self-reported smoking status from adult subjects. Although others have reported that smoking is negatively correlated with EoE risk, smoking is positively correlated with systemic oxidative stress (15–17). Thus, we cannot exclude smoking status as a potential confounder in our analysis of plasma GPX activity in adult EoE patients, which constitutes another limitation of the present study.

Despite known comorbidities, there remain patients with EoE but without other atopic diseases (and vice versa). Moreover, several targeted asthma therapies are ineffective for EoE. Together, these observations establish EoE as mechanistically related, yet unique from other atopic diseases. Though potentially underpowered, our findings that plasma GPX activity was reduced in pediatric patients with non-EoE atopic disease(s), but not EoE itself, further supports this distinction.

Additionally, atopic diseases (including EoE) manifest and progress differently in children than in adults. Broadly speaking, children tend to present with more acute, systemic symptoms, whereas adults tend to present with more chronic, localized symptoms. Furthermore, childhood-onset atopic disease often remits by adulthood. Thus, the natural progression of atopic disease from childhood into adulthood may underlie our inability to detect differences in plasma GPX activity in adults with and without non-EoE atopic disease.

Lastly, EoE, unlike other atopic conditions, is largely considered a tissue-restricted disease with few systemic manifestations. Namely, T helper 2-cell-mediated secretion of interleukin (IL)-4, IL-5, and IL-13, which lead to recruitment and activation of mast cells, eosinophils, and basophils, specifically occurs in the EoE esophagus, but not stomach and/or small intestine. Moreover, although some EoE patients exhibit elevated plasma immunoglobulin E (IgE) or blood *IL-5Rα* levels, IgE-targeted therapies for food allergy (omalizumab) and IL-5-/IL-5R*α*-targeted therapies for asthma (benralizumab, mepolizumab, reslizumab) have proven ineffective for EoE (18–21). In other words, the predominance of localized, rather than systemic, immunoinflammatory responses in EoE may partially explain our inability to detect differences in plasma GPX activity, even though esophageal GPX3 expression is reduced in EoE. By corollary, decreased plasma GPX activity in non-EoE atopic diseases may reflect systemic atopic inflammation, not specific atopic condition(s). Nevertheless, other groups have identified plasma/serum biomarkers for EoE, although none are antioxidant enzymes (6, 22, 23). Thus, identification of antioxidant EoE biomarkers may require direct sample collection from the esophagus.

In conclusion, while plasma GPX activity may not help identify EoE across ages, it may serve as a biomarker for atopic inflammation, particularly in pediatric patients. Future research efforts should incorporate esophagus-specific sampling methods to interrogate the mechanisms underlying these differences.

## Data Availability

The raw data supporting the conclusions of this article will be made available by the authors, without undue reservation.
