# Supplementary material for: Plasma glutathione peroxidase activity negatively correlates with atopic diseases in children but not adults: an exploratory study
Source: Front Allergy. 2026 Mar 12;7:1771105. doi: 10.3389/falgy.2026.1771105 (PMC13017957; doi:10.3389/falgy.2026.1771105)
Supplement: Supplementary file 1 [file Datasheet1.pdf]

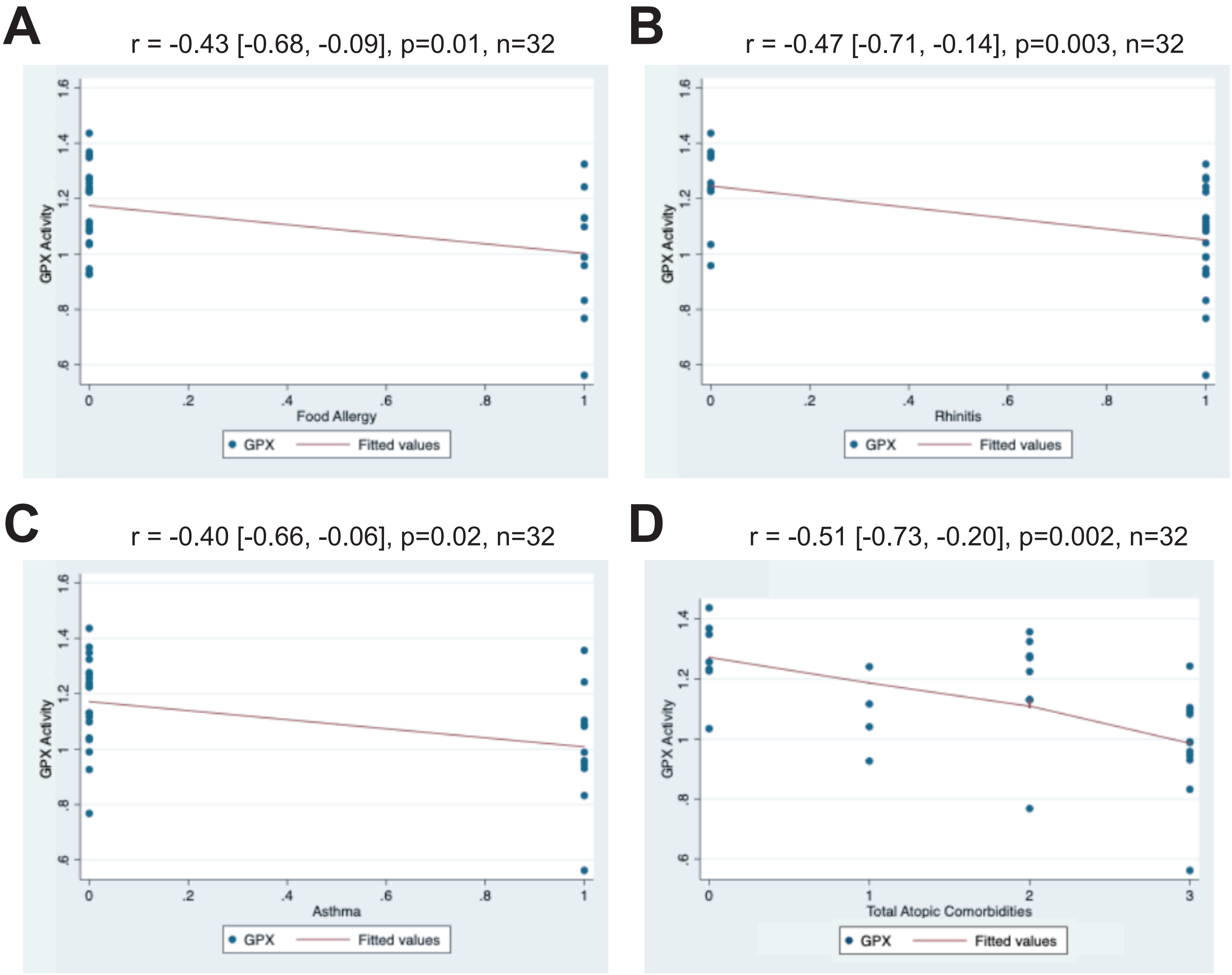

Supplementary Figure 1. Correlation plots of plasma GPX activity versus (A) food allergy, (B) allergic rhinitis, (C) asthma, or (D) total atopic burden.
